# Global Christianity and the Control of Its Neglected Tropical Diseases

**DOI:** 10.1371/journal.pntd.0003135

**Published:** 2014-11-20

**Authors:** Peter J. Hotez

**Affiliations:** 1 National School of Tropical Medicine and Departments of Pediatrics and Molecular Virology and Microbiology, Baylor College of Medicine, Houston, Texas, United States of America; 2 Sabin Vaccine Institute and Texas Children's Hospital Center for Vaccine Development, Houston, Texas, United States of America; 3 Department of Biology, Baylor University, Waco, Texas, United States of America; 4 James A Baker IIII Institute for Public Policy, Rice University, Houston, Texas, United States of America

*Revelations that the majority of the world's two billion Christians currently live in developing countries may have important implications for global neglected tropical disease control and elimination initiatives.*

**Figure 1:**
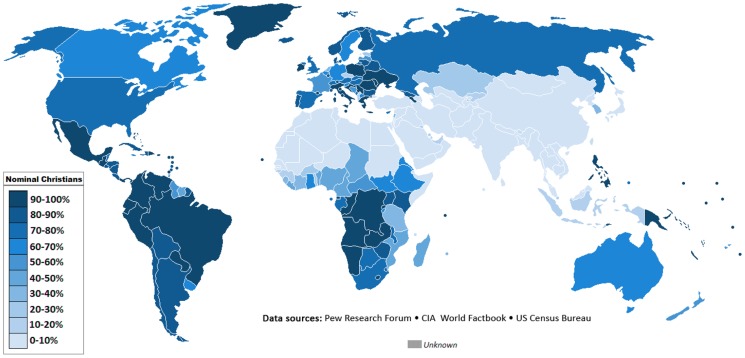
Christianity—percentage by country (2010). http://en.wikipedia.org/wiki/File:Christian_distribution.png.

In December 2011, the Pew Research Center's Forum on Religion & Public Life produced a landmark report titled “Global Christianity: A Report on the Size and Distribution of the World's Christian Population” [1]. Among the report's most important findings was that while the percentage of the world's population who are Christian has remained about the same over the last 100 years—in both 1910 and 2010, Christians comprised about one-third of the world's population—the geography of Christianity has changed dramatically [1]. In 1910, two-thirds of the world's Christians lived in Europe, whereas today only one-quarter are Europeans [1]. In place of Europe and due to population growth and expansion of Christianity in developing countries, more Christians than ever now live in Latin America, sub-Saharan Africa, and Asia. Indeed, of the ten countries with the largest number of Christians, seven are middle- or low-income countries, including Brazil (175.8 million), Mexico (107.8 million), the Philippines (86.8 million), Nigeria (80.5 million), China (67.1 million), the Democratic Republic of Congo (63.1 million), and Ethiopia (52.6 million) (Figure 1) [1]. Today, the Pew Forum reports that 61% of the world's Christians—about 1.3 billion people—live in the “Global South,” referring to Africa, Asia, and Latin America [1].

Prior to the Pew Forum's findings, I reported on an unexpectedly high burden of disease resulting from neglected tropical diseases in Catholic-majority countries [2]. If we now pair the Pew Forum numbers on global Christianity with new information released by the World Health Organization (WHO) and other sources on neglected tropical diseases (NTDs) [3]–[10], an astonishing picture of disease and poverty in Christendom emerges.

Shown in Table 1 are the largest Christian populations ranked among those that live in developing countries and areas. At the top of the list are Christians living in the Latin American and Caribbean (LAC) region; followed by the Philippines and China in Asia; and Nigeria, the Democratic Republic of Congo, Ethiopia, South Africa, and Kenya in sub-Saharan Africa. In all, eight regions are shown, and all except China are classified by the Pew Forum as Christian-majority regions. Together, almost one billion Christians live in these eight regions and comprise approximately three-quarters of the world's Christians from developing countries. While many of the Christians living in the areas listed in Table 1 are identified as Catholics (especially in the LAC countries), the Pew Forum study finds that in both Asia and sub-Saharan Africa, Protestants actually outnumber Catholics [1]. Indeed, outside the United States, Nigeria has the largest number of Protestants in the world (59.7 million), followed by China (58.0 million), Brazil (40.5 million), and South Africa (36.6 million) [1].

**Table 1:** NTDs and global christianity.

Country or Region (and Rank)	Estimated 2010 Christian Population [1]	Children Requiring Treatment for Intestinal Helminth Infections in 2012 [3], [4]	Population Requiring Treatment for Schistosomiasis in 2012 [5], [6]	Chagas Disease Cases [7]	Gambian Human African Sleeping Sickness in 2012 [8]
Latin American and Caribbean (LAC) region (1)	533.9 million^1^	49.3 million	1.6 million	7.5 million	None
Philippines (2)	86.8 million	31.2 million	0.5 million	None	None
Nigeria (3)	80.5 million	65.4 million	60.6 million	None	2
China (4)	67.1 million	25.6 million	0.1 million	None	None
DR Congo (5)	63.1 million	29.2 million	18.0 million	None	5,983
Ethiopia (6)	52.6 million	32.3 million	22.1 million	None	None
South Africa (7)	40.6 million	3.2 million	5.2 million	None	None
Kenya (8)	34.3 million	16.7 million	11.8 million	None	None
Total	958.9	252.9 million	119.9 million	7.5 million	5,985
Global	7.2 billion	875.9 million	249.4 million	7.8 million	7,106–7,216^2^
% in Leading Christian countries	13%	29%	48%	96%	83–84%

1 Determined by subtracting the Christian populations of the United States (246.8 million) and Canada (23.4 million) from the total number of Christians living in the Americas (804.1 million).

2 Two different numbers provided at www.who.int.

The latest information from WHO's Preventive Chemotherapy and Transmission Control (PCT) Databank allows us to superimpose onto the Pew Forum findings the number people who require treatment for either intestinal helminth infections [3], [4] or schistosomiasis [5], [6] (also shown in Table 1). Almost one-third of the world's children who require regular deworming for intestinal helminth infections and one-half of the world's population requiring schistosomiasis chemotherapy live in the eight leading regions where Christians live. Yet only about 13% of the world's population lives in these countries.

For Chagas disease—predominantly a disease of the LAC region, which is overwhelmingly Christian—almost all of the cases (96%) are found in Latin American nations [7]. Moreover, the Christian-majority nation of the Democratic Republic of Congo accounts for more than 80% of the world's cases of the Gambian form of human African trypanosomiasis (HAT) [8], [9].

The findings confirm that approximately one billion Christians who live in developing countries of Africa, Asia, and the Americas are highly vulnerable to NTDs. These diseases, which are highly destabilizing and associated with chronic and debilitating effects, represent a major force that traps the world's poorest Christians in poverty.

Today, both the intestinal helminth infections and schistosomiasis are being targeted through regular and periodic mass drug administration (“preventive chemotherapy”), costing less than 50 cents per person; but WHO estimates that currently only 35% of the world's population eligible for preventive chemotherapy actually receives essential NTD medicines [10]. Furthermore, less than one percent of people living with Chagas disease are treated with an antiparasitic agent [11]. In contrast, through case detection and management it may be feasible to eliminate the Gambian form of HAT in the coming years [12].

Christian institutions and organizations could have an important role in expanding the control or treatment of NTDs among the 1.3 billion Christians estimated to live in the Global South. As was pointed out earlier, both local archdioceses of the Catholic Church and Catholic charities have been involved in implementing deworming and other preventive chemotherapy strategies [2], but there are opportunities for the Vatican to have an expanded role particularly because of the unique commitment by Pope Francis to the poor. Similarly, the leadership of Orthodox Christianity has opportunities to speak out about NTDs, especially in a disease-endemic country such as Ethiopia. Many faith-based organizations could play an essential role in both preventive chemotherapy and programs related to health education and WASH (water, sanitation, and hygiene), while priests and pastors could serve as key advocates for enlisting community support for NTDs. Finally, the leading Christian-based universities in the United States and in developing countries could make important contributions on research and development for new or improved interventions to combat NTDs, such as new drugs, diagnostics, and vaccines, and help lead relevant programs of operational research. Christian organizations and institutions could help raise funds for such research.

Today, the NTDs represent some of the most common afflictions of global Christianity. It is especially noteworthy that Chagas disease and HAT are today almost exclusively a disease of impoverished Christians. Through NTDs, a renewed dialogue with faith-based organizations that work in developing countries and elements of the hierarchy of the Christian Church could make an important difference in global Christianity and the lives of the world's poorest people.
